# Engineering the future of advanced therapy medicinal products: a bioengineering call to action

**DOI:** 10.3389/fbioe.2026.1868007

**Published:** 2026-07-08

**Authors:** Giulia Nieri, Anna Spiller, Federico Stella, Alessandro Aiuti, Lorenzo Piemonti

**Affiliations:** 1 Biomedical Engineering Association (BEA), Politecnico di Milano, Milan, Italy; 2 Hematology Division, Fondazione IRCCS Istituto Nazionale dei Tumori, Milan, Italy; 3 San Raffaele Telethon Institute for Gene Therapy (SR-TIGET), IRCCS San Raffaele Scientific Institute, Milan, Italy; 4 Università Vita-Salute San Raffaele, Milan, Italy; 5 Diabetes Research Institute (DRI), IRCCS San Raffaele Scientific Institute, Milan, Italy

**Keywords:** advanced therapy medicinal products, biomanufacturing, biomaterials, car-t, gene therapy, health equity, iPSC-derived cells, process analytical technology

## Abstract

Advanced Therapy Medicinal Products — cell therapies, gene therapies, and tissue-engineered products — are beginning to deliver on the promise of curative medicine: CAR-T therapies double survival in chemotherapy-refractory lymphomas, gene therapies reverse the natural history of spinal muscular atrophy and hemoglobinopathies, and Pluripotent Stem Cell (PSC)-derived islet transplantation renders type 1 diabetic patients insulin-independent. Yet the trajectory from proof-of-concept to equitable, scalable deployment is consistently impeded not only by unresolved biology but also by engineering, manufacturing, logistical, regulatory, and economic bottlenecks that the bioengineering community has not engaged with at the required scale. In this Perspective, grounded in clinical experience across hematological malignancies, monogenic diseases, and metabolic disorders, we identify five rate-limiting bottlenecks where bioengineering intervention is urgently needed and uniquely tractable: scalable and adaptive biomanufacturing; real-time in-process quality control; precise targeted delivery; biomaterial and scaffold engineering for cellular engraftment and immune protection; and data-driven patient stratification constrained by health equity. We argue that the evolving regulatory landscape in Europe — including the European Biotech Act framework and ICH Quality by Design principles — creates structural incentives for engineering-led solutions, and that economic sustainability requires bioengineering to drive down production costs and enable the off-the-shelf transition. We call on the bioengineering community to engage with ATMP translation not as technical support to clinical medicine, but as a constitutive partner shaping its pace, cost, and equity.

## 1 Introduction

The conceptual foundations of ATMPs span five decades: tumor-infiltrating lymphocyte therapy (Rosenberg et al., 1988; Rohaan et al., 2022), graft-versus-leukemia effect in allogeneic stem cell transplantation (Horowitz et al., 1990), somatic gene correction (Blaese et al., 1995), and chimeric antigen receptor design (Sadelain et al., 2013). Today these concepts have matured into approved products with measurable clinical impact: CD19-targeted CAR-T achieves >50% overall survival in chemotherapy-refractory large B-cell lymphoma (Locke et al., 2019; Schuster et al., 2019; Abramson et al., 2020) versus ∼20% with salvage regimens (Crump et al., 2017); AAV-mediated gene replacement transforms spinal muscular atrophy type 1 from uniformly fatal to compatible with normal neurodevelopment (Mendell et al., 2017); lentiviral gene therapy restores transfusion independence in beta-thalassemia (Thompson et al., 2018); and PSC-derived islet transplantation has achieved insulin independence in initial clinical cohorts of type 1 diabetes (Wang et al., 2024; Reichman et al., 2025). The administration of a bespoke single-patient gene therapy designed and manufactured within months from diagnosis (Kim et al., 2019) marks the outer frontier of individualized medicine.

**FIGURE 1:**
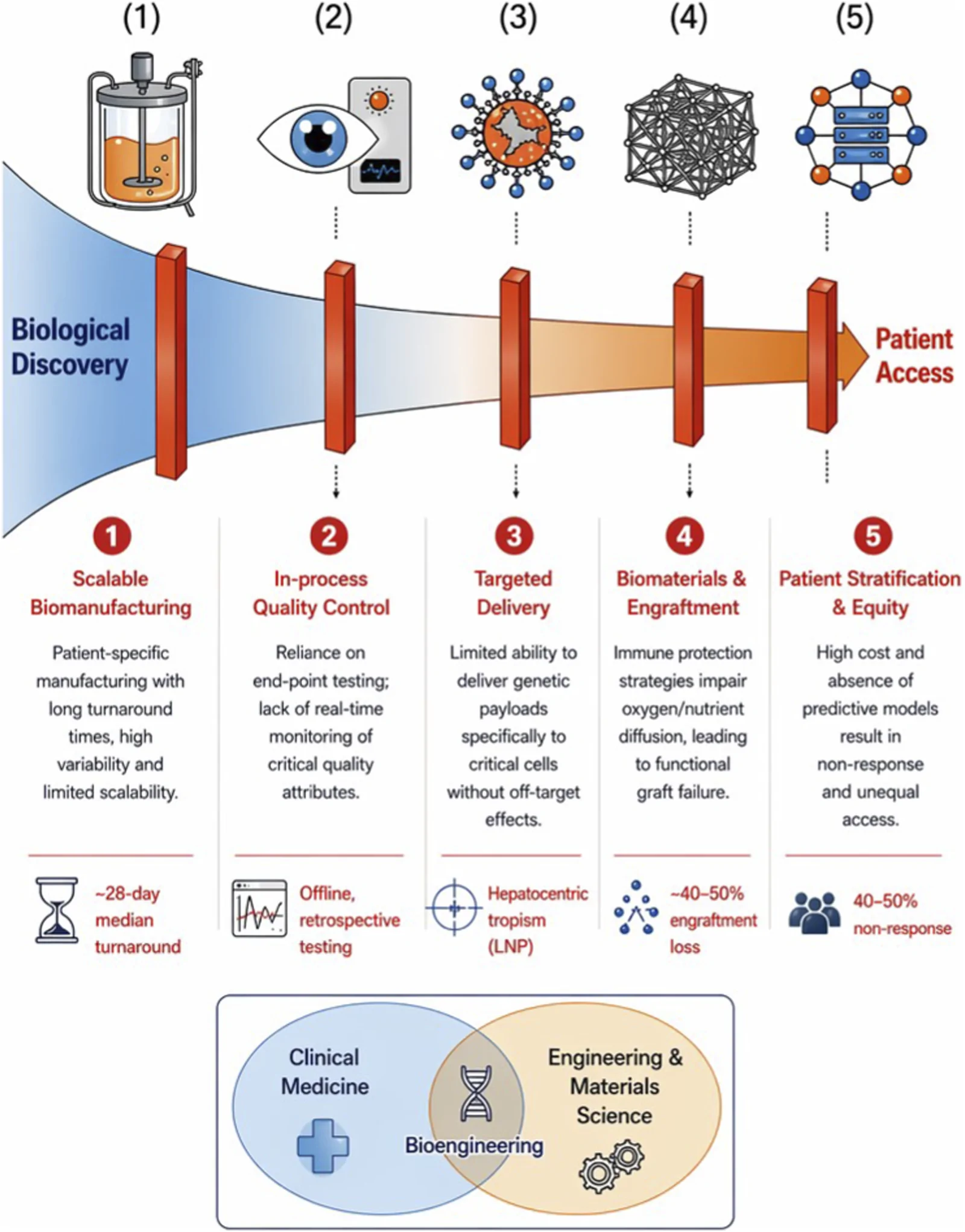
The ATMP translation gap: five engineering bottlenecks between biological discovery and equitable patient access. Each barrier narrows the therapeutic pipeline. Inset: bioengineering occupies the overlap between clinical medicine and engineering/materials science. CQA, critical quality attribute; LNP, lipid nanoparticle.

Against these milestones, the translation gap is stark: ∼40,000 patients have received approved CAR-T globally since 2017, representing a fraction of those eligible, with estimates suggesting that fewer than 20% of eligible patients ultimately receive this treatment (Singh et al., 2026; Borgert, 2021); gene therapies carry price tags of €300,000–€1,000,000+, inaccessible to most health systems (Han et al., 2026); pancreatic islet transplantation required 20–30 years from first clinical use to approved standard-of-care status (Catarinella et al., 2025; Marfil-Garza et al., 2022). The dominant narrative attributes these limitations to biological complexity and regulatory necessity. We argue this framing is incomplete. The most tractable rate-limiting bottlenecks are problems of process design, measurement science, materials engineering, and algorithmic decision support — domains where bioengineering is uniquely competent and systematically underdeployed. This Perspective maps five such bottlenecks (Figure 1) and calls for the bioengineering community to engage with them at the scale and ambition the clinical need demands.

## 2 Bottleneck 1 — scalable and adaptive biomanufacturing

### 2.1 Inverting the industrial paradigm

The pharmaceutical manufacturing paradigm — produce an identical molecule at scale and reduce marginal cost through volume — is structurally incompatible with autologous ATMPs. CAR-T therapies, patient-matched iPSC-derived products, and individualized gene therapies require a *batch-of-one* model: each product originates from biologically variable patient material, requires independent release testing, and has a narrow viability window. Marginal cost does not decline with volume; the economic engine of conventional drug manufacturing does not apply.

Current GMP infrastructure compounds the problem. Release testing of a living cellular product — multi-parameter flow cytometry, functional potency assays, residual pluripotency testing, sterility — spans days to weeks during which product quality degrades. Centralized autologous CAR-T production involves median turnaround of ∼28 days from leukapheresis to infusion-ready product. This is precisely the type of problem that process systems engineering (PSE) was developed to address in other manufacturing domains. PSE provides a formal toolkit — supervisory process control, dynamic scheduling, real-time optimization, and digital-twin modeling of individual unit operations — for managing processes that are variable, time-constrained, and tightly coupled. Applied to autologous CAR-T, this framework reframes the manufacturing challenge from one of artisanal repetition to one of system-level design and control, offering a structured route to reduce turnaround time, contain batch-to-batch variability, and increase throughput without compromising product quality (Wayne, 2026). The translation of PSE methods from chemical and continuous-manufacturing contexts to the living, donor-dependent processes of cell therapy is itself a bioengineering research frontier. Biological variability in starting material — cells from pre-treated, elderly, or immunocompromised patients differ systematically from healthy donors — propagates through the process. Growth factor lot-to-lot variability (EGF, FGF2, Activin A) spans 10%–30% even between GMP-certified suppliers, directly affecting differentiation yield. Unlike chemical synthesis, cell processes involve stochastic gene expression and environmental sensitivities — to shear stress, oxygen gradients, substrate rigidity — that standardization alone cannot eliminate.

Automation of a poorly characterized process produces automated variability. What is required is adaptive process control: closed-system platforms integrating PAT sensors with machine vision and metabolomic readouts, dynamically adjusting parameters in response to real-time biological state. Computational fluid dynamics modeling of bioreactor geometry could enable rational scale-up design, replacing costly empirical optimization. Decentralized miniaturized platforms would reduce geographic inequity by bringing manufacturing closer to patients whose products have shelf lives measured in hours.

**FIGURE 2:**
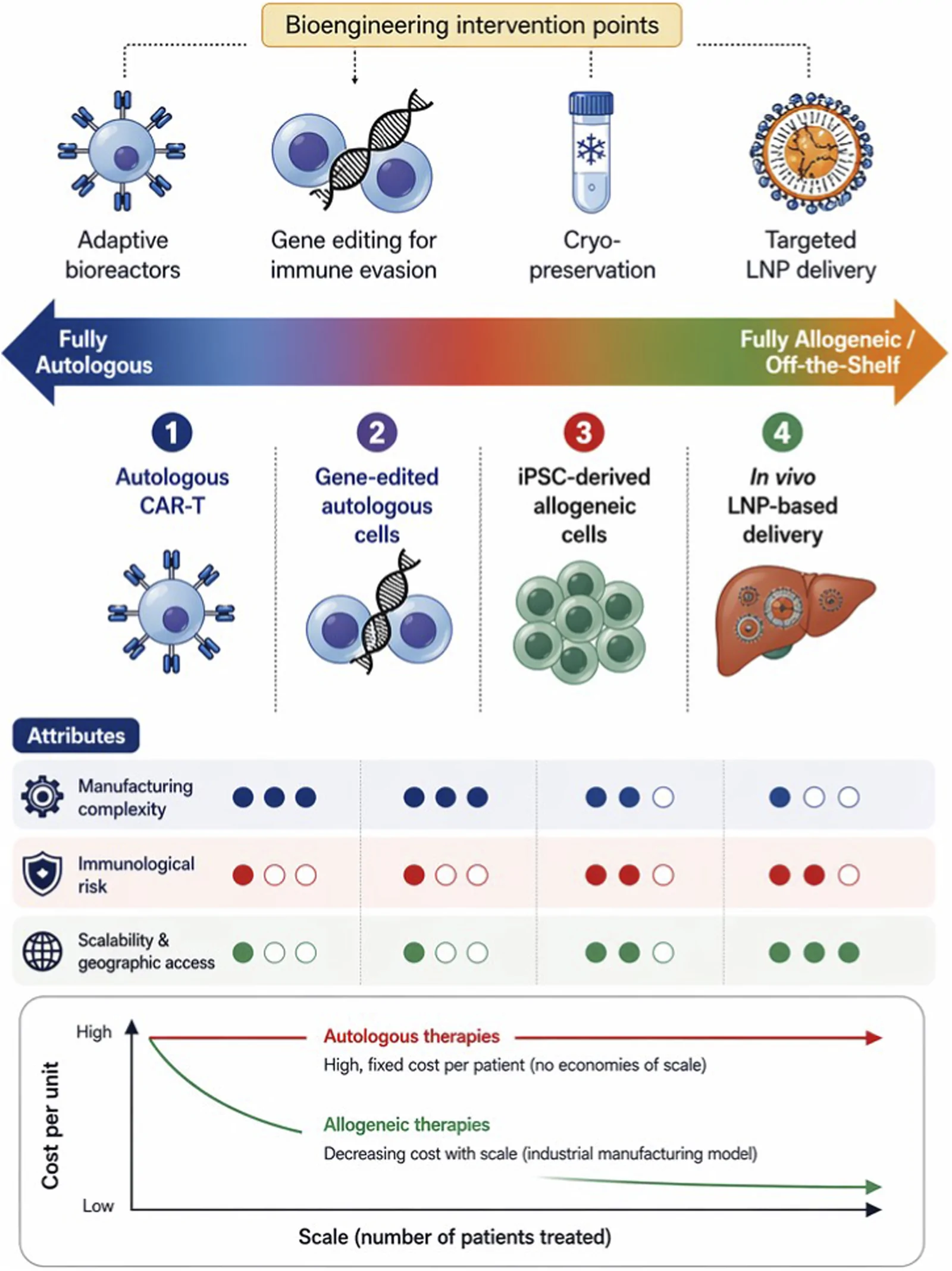
The autologous-to-allogeneic product spectrum and its engineering implications. Attribute ratings (manufacturing complexity, immunological risk, scalability/access) vary across four product archetypes. Right inset: cost-per-unit curves illustrate the economic rationale for the allogeneic transition. iPSC, induced pluripotent stem cell; LNP, lipid nanoparticle; HLA, human leukocyte antigen.

The strategic endpoint is off-the-shelf products — manufactured from standardized lines, cryo-preserved, distributed like conventional drugs — which recover the economics of scale. This requires upstream solutions: immune evasion engineering (Section 5), cryo-preservation protocol development, and line manufacturing standardization. Figure 2 maps the autologous-to-allogeneic spectrum and key bioengineering intervention points.

## 3 Bottleneck 2 — in-process quality control

### 3.1 From end-point testing to real-time process understanding

ATMP quality control is currently retrospective: manufacturing completes, end-of-process samples are tested, product is released or rejected. For a living autologous product, a failed release test is irreversible — it may directly deprive a deteriorating patient of their only therapeutic option.

The alternative — real-time process understanding per ICH Q8 Quality by Design — requires non-destructive, continuous monitoring of critical quality attributes (CQAs). For CAR-T, these include T-cell phenotypic composition (naive, stem cell memory, effector memory subsets with distinct *in vivo* persistence profiles), transduction efficiency, metabolic fitness, and exhaustion markers. For iPSC-derived products, absence of residual undifferentiated cells with tumorigenic potential is safety-critical. Current sensors monitor culture environment (O_2_, pH, temperature, glucose/lactate) — not the product itself. Machine vision systems can infer phenotypic identity from brightfield and fluorescence morphology (Moen et al., 2019); Raman and NIR spectroscopy enable non-destructive metabolite quantification (Luo et al., 2025); machine learning integration of heterogeneous in-process signals into Digital Twin models enables course-correction before batches are irretrievably compromised (Riezzo et al., 2025). The role of artificial intelligence, however, extends well beyond in-process quality control. AI methods are increasingly deployed across the entire pharmaceutical value chain — target identification and drug discovery, formulation development, manufacturing process optimization, automated quality control, and post-market surveillance — and the same paradigm shift is now reaching ATMP production (Huanbutta et al., 2024). For advanced therapies specifically, this breadth is significant: the same underlying modeling competencies that predict lot quality can, in principle, be redeployed upstream to optimize differentiation protocols and downstream to monitor long-term safety signals in treated patients. Realizing this potential requires bioengineers who can build models that are not only accurate but also transferable across the distinct stages of the value chain, each with its own data structures, regulatory constraints, and failure modes.

A critical unresolved dimension is regulatory: EMA and FDA lack clear pathways for qualifying adaptive AI-assisted algorithms within validated GMP processes. This gap between technical capability and regulatory acceptability requires engineers who can design GMP-compliant AI validation frameworks — demonstrating process equivalence, interpretability, and failure-mode containment — a role currently unfilled by any other discipline.

## 4 Bottleneck 3 — targeted delivery

### 4.1 Getting the right payload to the right cell

Gene therapy and *in vivo* cellular reprogramming require delivery of nucleic acid payloads — corrective transgenes, Clustered Regularly Interspaced Short Palindromic Repeats (CRISPR) complexes, base editors, therapeutic mRNAs — specifically to target cells at therapeutic intracellular concentrations without consequential off-target effects. Insufficient delivery means failure; non-specific delivery to dividing progenitors risks insertional mutagenesis.

Adeno-associated virus (AAV) vectors, produced at 200–400 L bioreactor scale, remain the dominant *in vivo* gene therapy platform but retain fundamental limitations: capsid tropism is incompletely redirectable; pre-existing humoral immunity excludes a significant patient fraction and precludes re-dosing; packaging capacity (∼4.7 kb) excludes large transgenes. AlphaFold2-enabled structure-guided engineering and directed evolution are generating capsid variants with improved selectivity and reduced immunogenicity (Deverman et al., 2016), but GMP-scale production and characterization of engineered capsids remains a bioprocess bottleneck.

Lipid nanoparticles (LNPs) — scalably produced, chemically defined, immunologically naïve — transformed mRNA vaccine delivery but currently exhibit predominantly hepatocentric biodistribution. Surface functionalization with cell-selective targeting ligands (antibody fragments, aptamers, receptor-binding peptides) to redirect LNP tropism is an active engineering frontier (Cheng et al., 2020). *In vivo* CAR-T generation via T-lymphocyte-targeted LNPs — delivering CAR-encoding mRNA to circulating T cells and bypassing *ex vivo* manufacturing — has entered early clinical investigation (An et al., 2026; Gao et al., 2026). The same platform logic applied to pancreatic progenitors, hematopoietic stem cells, or neurons could transform the scalability and equity of therapies currently dependent on complex *ex vivo* manipulation.

## 5 Bottleneck 4 — biomaterials and scaffold engineering

### 5.1 The microenvironment as therapeutic determinant

Cellular encapsulation for immune isolation exemplifies the distinction between a sound biological concept and an unsolved engineering problem. Semipermeable encapsulation consistently suppresses adaptive rejection in preclinical models but has failed to achieve durable therapeutic function in human trials (Keymeulen et al., 2024; Philpott, 2025). The failure mechanism is specific: barriers that exclude immune effectors impose diffusion limits restricting oxygen delivery. Insulin-secreting beta cells require oxygen tensions ∼10-fold higher for full biosynthetic function than for survival; cells in poorly vascularized capsules survive but produce insufficient insulin for glycemic control (Pham et al., 2025). The problem is not immunological design — it is oxygenation engineering within an immune-excluded space. Candidate solutions include oxygen-generating materials (solid peroxides) within devices, scaffold architectures promoting host vascularization through controlled pore geometry, and pro-tolerogenic biomaterial matrices incorporating anti-inflammatory cytokines or regulatory T-cell-recruiting signals.

Beyond encapsulation, ECM composition and mechanics are determinants of cell identity and function (Macri-Pellizzeri et al., 2018). Substrate rigidity (∼1–2 kPa for pancreatic tissue) influences iPSC-derived beta-cell functional maturation through mechanotransduction; 3D co-culture architectures reproducing islet niche interactions improve glucose-stimulated insulin secretion kinetics. Decellularized organ matrices — preserving vascular architecture and growth factor gradients — offer scalable biological scaffold sources. Rational design of synthetic or biological scaffolds recapitulating these properties with GMP-compatible reproducibility is a core bioengineering challenge.

Orthogonally, gene editing for immune evasion approaches the same problem from a molecular angle: HLA class I/II deletion eliminates T-cell recognition; CD47 overexpression suppresses NK-mediated killing (Hu et al., 2023; Carlsson et al., 2025; Hu et al., 2025). Early clinical results demonstrate months of allogeneic cell persistence without immunosuppression. This strategy requires engineering inducible suicide switches — activatable if malignant transformation occurs — adding complexity requiring regulatory qualification but representing a potentially definitive solution to the immunosuppression burden that currently limits ATMP access.

## 6 Bottleneck 5 — patient stratification and health equity

### 6.1 Making high-cost therapies equitably effective

ATMPs are the most expensive per-treatment interventions in pharmaceutical history: CAR-T at €300,000–€500,000; approved gene therapies exceeding €1,000,000. These prices reflect genuine upstream costs but also the absence of prospective responder identification systems. Non-responders receive burdensome ineffective treatment; potential responders may not survive the manufacturing window. Predictive stratification is simultaneously an economic efficiency, clinical safety, and equity problem.

CAR-T response is determined at multiple levels: tumor (antigen density, microenvironment immunosuppression), patient (prior treatment burden, T-cell repertoire fitness, systemic inflammation, and — increasingly recognized — the composition of the gut microbiota), and product (phenotypic composition — stem cell memory phenotypes associated with superior persistence versus terminally exhausted effectors predicting early failure) (Fraietta et al., 2018). Among these determinants, the gut microbiota has emerged as a particularly instructive example of a variable that is biologically influential yet absent from current predictive frameworks. Specific commensal taxa have been associated with CAR-T expansion, persistence, and the incidence of immune-related toxicities, suggesting that microbiome composition carries predictive — and potentially modifiable — information (Asokan et al., 2023). Its omission from stratification models is not a biological oversight but a measurement and data-integration gap: microbiome profiling is rarely standardized, rarely collected at the relevant timepoints, and rarely linked to manufacturing and outcome data in interoperable form. This exemplifies the broader engineering challenge of the section — that the value of a predictor is realized only when it can be measured reproducibly and integrated into a model that informs a clinical decision. No clinician integrates this data volume at the point of care. Multi-center real-world registries combined with machine learning could build composite predictive scores identifying patients unlikely to respond (who should access alternative trials), and patients whose disease velocity makes any manufacturing delay life-threatening (triggering bridge therapy or *in vivo* alternatives). This is fundamentally a bioengineering problem: model performance depends on standardized interoperable data collection — flow cytometric starting material profiles, product transcriptomics, imaging-based tumor burden — currently neither standardized nor connected across sites. Engineers designing data pipelines, measurement protocols, and interpretable clinical-decision models occupy an irreplaceable role.

Equity must be an explicit design constraint, not a *post hoc* consideration. Models trained on historically treated populations inherit their selection biases — geographic, socioeconomic, demographic — systematically disadvantaging underserved patients (Obermeyer et al., 2019). Proactive algorithmic fairness, domain adaptation, and representative data collection are engineering obligations. Equally, the clinicians and data scientists who deploy these models require structured training in their assumptions, failure modes, and boundaries of applicability: an algorithm used without interpretive competence compounds rather than corrects existing inequities. More fundamentally, every choice between autologous and allogeneic, centralized and decentralized, viral vector and LNP directly determines who can access a therapy and at what cost — engineering decisions with irreducible ethical weight.

## 7 Regulatory and economic context

Engineering solutions must navigate a framework designed for conventional pharmaceuticals. EC Regulation 1394/2007 established the ATMP category and centralized EMA evaluation, but its limitations — slow classification, inconsistent hospital exemption across member states, linear dossier submission — impede innovation. The European Biotech Act (proposed December 2025), which amends the ATMP Regulation (EC) No 1394/2007 and the Clinical Trials Regulation, would introduce accelerated regulatory pathways for advanced therapies - including faster clinical-trial authorization (the FAST-EU pilot) - formalized rolling review enabling early regulatory feedback, moves toward a harmonized hospital exemption, and conditional authorization linking reimbursement to mandatory real-world registries. The ICH Q8/Q10 Quality by Design framework, if fully implemented for ATMPs, creates regulatory incentives for the in-process sensing investments described in Section 3, making the regulatory and engineering agendas convergent rather than adversarial.

Economically, one-time curative interventions are incommensurable with chronic-drug reimbursement models. Outcome-based payment and multi-year amortization agreements are emerging but require data infrastructure — standardized outcome registries, long-term follow-up systems — that bioengineering must help build. Non-profit ATMP development structures with public funding and accessible IP licensing (exemplified by CIRM and IHI) address the structural tension between private investment logic and the public-good character of curative rare disease therapies, but their scaling requires the manufacturing cost reductions that only engineering innovation can deliver.

## 8 Discussion

The clinical advances described are genuine and consequential, achieved by investigators crossing disciplinary boundaries over timescales measured in decades. What distinguished faster translation trajectories — SMA gene therapy, CD19 CAR-T — was not superior biology but more effective engineering: scalable manufacturing platforms, reproducible vector production, regulatory strategies anticipating agency expectations. Islet transplantation’s 20–30 years path to approval illustrates the cost of the opposite.

We do not claim engineering alone determines success. Biological unknowns remain substantial: iPSC-derived beta-cell functional immaturity mechanisms are unresolved; determinants of durable CAR-T persistence are incompletely characterized; optimal immune tolerance strategies for allogeneic products are contested. Premature engineering optimization of poorly understood biology produces elegant solutions to wrong problems — the encapsulation history is a cautionary example.

Our claim is that the interface between biological understanding and engineering implementation is the primary rate-limiting step in ATMP translation today, and is systematically underinvested. The clinician identifying why encapsulated beta cells survive but fail to control glycemia has defined an oxygenation engineering specification. The hematologist observing 40%–50% CAR-T non-response despite adequate starting material has defined a process characterization problem. The gene therapist unable to re-dose due to AAV immunogenicity has defined a delivery engineering problem. Clinical observation, in each case, generates the engineering requirement. The missing element is the bioengineering interlocutor fluent in both languages — able to receive a clinical failure mode and translate it into tractable engineering design requirements.

Forming such interlocutors is an educational challenge. Bioengineering programs incorporating clinical immersion, regulatory science, and bioethics; research structures co-locating clinical and engineering teams with shared translation accountability: these are institutional responses to a structural gap that no amount of *post hoc* collaboration can fully bridge. Integrated medicine–biomedical engineering curricula already exist — in Italy, notably, within a single private institution — but remain isolated experiments rather than a systemic response. Scaling and replicating these models across public universities, and embedding them within the research consortia driving ATMP development, is an investment whose returns would be measured in accelerated translation and reduced inequity.

We close with a dual call. Specific: the five bottlenecks mapped here — adaptive biomanufacturing, real-time quality control, targeted delivery, biomaterial microenvironment engineering, and equity-aware patient stratification — are tractable engineering problems with defined clinical requirements, large unmet need, and insufficient engineering attention. General: every technical choice in ATMP development — autologous or allogeneic, centralized or decentralized, viral or non-viral — carries ethical weight proportional to its direct impact on patient access. The biology has shown what is possible. Whether it becomes accessible — equitably, durably, at scale — depends on what engineers build next.

## Data Availability

The original contributions presented in the study are included in the article/supplementary material, further inquiries can be directed to the corresponding author.

## References

[B1] Abramson J. S. Palomba M. L. Gordon L. I. Lunning M. A. Wang M. Arnason J. (2020). Lisocabtagene maraleucel for patients with relapsed or refractory large B-Cell lymphomas (transcend nhl 001): a multicentre seamless design study. Lancet 396 (10254), 839–852. 10.1016/s0140-6736(20)31366-0 32888407

[B2] An N. Wang D. Zhang P. Zhang J. Parone P. Hu J. (2026). *In vivo* generation of anti-bcma Car-T cells in relapsed or refractory multiple myeloma: a phase 1 study. Nat. Med. 32 (4), 1257–1266. 10.1038/s41591-026-04244-6 41882404

[B3] Asokan S. Cullin N. Stein-Thoeringer C. K. Elinav E. (2023). Car-T cell therapy and the gut microbiota. Cancers (Basel) 15 (3), 794. 10.3390/cancers15030794 36765752 PMC9913364

[B4] Blaese R. M. Culver K. W. Miller A. D. Carter C. S. Fleisher T. Clerici M. (1995). T lymphocyte-directed gene therapy for Ada- scid: initial trial results after 4 years. Science 270 (5235), 475–480. 10.1126/science.270.5235.475 7570001

[B5] Borgert R. (2021). Improving outcomes and mitigating costs associated with car T-Cell therapy. Am. J. Manag. Care 27 (13), 253–261. 10.37765/ajmc.2021.88737 34407361

[B6] Carlsson P. O. Hu X. Scholz H. Ingvast S. Lundgren T. Scholz T. (2025). Survival of transplanted allogeneic beta cells with no immunosuppression. N. Engl. J. Med. 393 (9), 887–894. 10.1056/NEJMoa2503822 40757665

[B7] Catarinella D. Melzi R. Mercalli A. Magistretti P. Tentori S. Gremizzi C. (2025). Long-term outcomes of pancreatic islet transplantation alone in type 1 diabetes: a 20-Year single-centre study in Italy. Lancet Diabetes and Endocrinol. 13 (4), 279–293. 10.1016/S2213-8587(24)00341-3 39929222

[B8] Cheng Q. Wei T. Farbiak L. Johnson L. T. Dilliard S. A. Siegwart D. J. (2020). Selective organ targeting (sort) nanoparticles for tissue-specific mrna delivery and crispr-cas gene editing. Nat. Nanotechnol. 15 (4), 313–320. 10.1038/s41565-020-0669-6 32251383 PMC7735425

[B9] Crump M. Neelapu S. S. Farooq U. Van Den Neste E. Kuruvilla J. Westin J. (2017). Outcomes in refractory diffuse large B-Cell lymphoma: results from the international Scholar-1 study. Blood 130 (16), 1800–1808. 10.1182/blood-2017-03-769620 28774879 PMC5649550

[B10] Deverman B. E. Pravdo P. L. Simpson B. P. Kumar S. R. Chan K. Y. Banerjee A. (2016). Cre-dependent selection yields aav variants for widespread gene transfer to the adult brain. Nat. Biotechnol. 34 (2), 204–209. 10.1038/nbt.3440 26829320 PMC5088052

[B11] Fraietta J. A. Lacey S. F. Orlando E. J. Pruteanu-Malinici I. Gohil M. Lundh S. (2018). Determinants of response and resistance to Cd19 chimeric antigen receptor (Car) T cell therapy of chronic lymphocytic leukemia. Nat. Med. 24 (5), 563–571. 10.1038/s41591-018-0010-1 29713085 PMC6117613

[B12] Gao P. Liu J. Ma J. Ma H. Zhang Y. (2026). *In vivo* Car-T cell therapy: latest updates from 2025 ash annual meeting. J. Hematol. and Oncol. 19 (1), 21. 10.1186/s13045-026-01787-6 41957634 PMC13063456

[B13] Han Y. Andreoletti M. Minssen T. Vayena E. Ormond K. E. (2026). The impacts of pricing and reimbursement policies on access to cell and gene therapies across Europe. J. Community Genet. 17 (1), 23. 10.1007/s12687-026-00860-4 41582289 PMC12832597

[B14] Horowitz M. M. Gale R. P. Sondel P. M. Goldman J. M. Kersey J. Kolb H. J. (1990). Graft-Versus-leukemia reactions after bone marrow transplantation. Blood 75 (3), 555–562. 10.1182/blood.V75.3.555.555 2297567

[B15] Hu X. White K. Olroyd A. G. DeJesus R. Dominguez A. A. Dowdle W. E. (2023). Hypoimmune induced pluripotent stem cells survive long term in fully immunocompetent, allogeneic rhesus macaques. Nat. Biotechnol. 42, 413–423. 10.1038/s41587-023-01784-x 37156915 PMC10940156

[B16] Hu X. Beauchesne P. Wang C. Wong A. Deuse T. Schrepfer S. (2025). Hypoimmune Cd19 car T cells evade allorejection in patients with cancer and autoimmune disease. Cell Stem Cell 32 (9), 1356–1368. 10.1016/j.stem.2025.07.009 40812299

[B17] Huanbutta K. Burapapadh K. Kraisit P. Sriamornsak P. Ganokratanaa T. Suwanpitak K. (2024). Artificial intelligence-driven pharmaceutical industry: a paradigm shift in drug discovery, formulation development, manufacturing, quality control, and post-market surveillance. Eur. J. Pharm. Sci. 203, 106938. 10.1016/j.ejps.2024.106938 39419129

[B18] Keymeulen B. De Groot K. Jacobs-Tulleneers-Thevissen D. Thompson D. M. Bellin M. D. Kroon E. J. (2024). Encapsulated stem cell–derived Β cells exert glucose control in patients with type 1 diabetes. Nat. Biotechnol. 42 (10), 1507–1514. 10.1038/s41587-023-02055-5 38012450 PMC11471599

[B19] Kim J. Hu C. Moufawad El Achkar C. Black L. E. Douville J. Larson A. (2019). Patient-customized oligonucleotide therapy for a rare genetic disease. N. Engl. J. Med. 381 (17), 1644–1652. 10.1056/NEJMoa1813279 31597037 PMC6961983

[B20] Locke F. L. Ghobadi A. Jacobson C. A. Miklos D. B. Lekakis L. J. Oluwole O. O. (2019). Long-term safety and activity of axicabtagene ciloleucel in refractory large B-Cell lymphoma (Zuma-1): a single-arm, multicentre, phase 1-2 trial. Lancet Oncol. 20 (1), 31–42. 10.1016/s1470-2045(18)30864-7 30518502 PMC6733402

[B21] Luo Z. Chen H. Bi X. Ye J. (2025). Monitoring kinetic processes of drugs and metabolites: surface-enhanced raman spectroscopy. Adv. Drug Deliv. Rev. 217, 115483. 10.1016/j.addr.2024.115483 39675433

[B22] Macri-Pellizzeri L. De-Juan-Pardo E. M. Prosper F. Pelacho B. (2018). Role of substrate biomechanics in controlling (stem) cell fate: implications in regenerative medicine. J. Tissue Eng. Regen. Med. 12 (4), 1012–1019. 10.1002/term.2586 29024545

[B23] Marfil-Garza B. A. Imes S. Verhoeff K. Hefler J. Lam A. Dajani K. (2022). Pancreatic islet transplantation in type 1 diabetes: 20-year experience from a single-centre cohort in Canada. Lancet Diabetes Endocrinol. 10 (7), 519–532. 10.1016/s2213-8587(22)00114-0 35588757

[B24] Mendell J. R. Al-Zaidy S. Shell R. Arnold W. D. Rodino-Klapac L. R. Prior T. W. (2017). Single-dose gene-replacement therapy for spinal muscular atrophy. N. Engl. J. Med. 377 (18), 1713–1722. 10.1056/NEJMoa1706198 29091557

[B25] Moen E. Bannon D. Kudo T. Graf W. Covert M. Van Valen D. (2019). Deep learning for cellular image analysis. Nat. Methods 16 (12), 1233–1246. 10.1038/s41592-019-0403-1 31133758 PMC8759575

[B26] Obermeyer Z. Powers B. Vogeli C. Mullainathan S. (2019). Dissecting racial bias in an algorithm used to manage the health of populations. Science 366 (6464), 447–453. 10.1126/science.aax2342 31649194

[B27] Pham T. T. Tran P. L. Tempelman L. A. Stone S. G. Piccirillo C. Li A. (2025). A continuously oxygenated macroencapsulation system enables high-density packing and delivery of insulin-secreting cells. Nat. Commun. 16 (1), 7199. 10.1038/s41467-025-62271-2 40789835 PMC12339981

[B28] Philpott J. (2025). Vertex Halts Development of Diabetes Cell Therapy After Trial Failure. Available online at: https://www.clinicaltrialsarena.com/news/vertex-halts-development-of-diabetes-cell-therapy-after-trial-failure/ (Accessed June 27 2026).

[B29] Reichman T. W. Markmann J. F. Odorico J. Witkowski P. Fung J. J. Wijkstrom M. (2025). Stem cell-derived, fully differentiated islets for type 1 diabetes. N. Engl. J. Med. 393 (9), 858–868. 10.1056/NEJMoa2506549 40544428

[B30] Riezzo L. Kay H. Feng Y. Jing K. Zhang D. (2025). Accelerating bioprocess digital twin development by integrating hybrid modelling with transfer learning. Chem. Eng. J. 511, 162018. 10.1016/j.cej.2025.162018

[B31] Rohaan M. W. Borch T. H. van den Berg J. H. Met Ö. Kessels R. Geukes Foppen M. H. (2022). Tumor-infiltrating lymphocyte therapy or ipilimumab in advanced melanoma. N. Engl. J. Med. 387 (23), 2113–2125. 10.1056/NEJMoa2210233 36477031

[B32] Rosenberg S. A. Packard B. S. Aebersold P. M. Solomon D. Topalian S. L. Toy S. T. (1988). Use of tumor-infiltrating lymphocytes and Interleukin-2 in the immunotherapy of patients with metastatic melanoma. A preliminary report. N. Engl. J. Med. 319 (25), 1676–1680. 10.1056/nejm198812223192527 3264384

[B33] Sadelain M. Brentjens R. Rivière I. (2013). The basic principles of chimeric antigen receptor design. Cancer Discov. 3 (4), 388–398. 10.1158/2159-8290.Cd-12-0548 23550147 PMC3667586

[B34] Schuster S. J. Bishop M. R. Tam C. S. Waller E. K. Borchmann P. McGuirk J. P. (2019). Tisagenlecleucel in adult relapsed or refractory diffuse large B-Cell lymphoma. N. Engl. J. Med. 380 (1), 45–56. 10.1056/NEJMoa1804980 30501490

[B35] Singh S. Greulich C. Perez A. Terrell K. Walz-Jensen J. Dalzell M. D. (2026). Operationalizing access for chimeric antigen receptor T cell therapies: a cross-functional perspective. Mayo Clin. Proc. Innov. Qual. Outcomes 10 (1), 100682. 10.1016/j.mayocpiqo.2025.100682 41488602 PMC12756569

[B36] Thompson A. A. Walters M. C. Kwiatkowski J. Rasko J. E. J. Ribeil J. A. Hongeng S. (2018). Gene therapy in patients with transfusion-dependent Β-Thalassemia. N. Engl. J. Med. 378 (16), 1479–1493. 10.1056/NEJMoa1705342 29669226

[B37] Wang S. Du Y. Zhang B. Meng G. Liu Z. Liew S. Y. (2024). Transplantation of chemically induced pluripotent stem-cell-derived islets under abdominal anterior rectus sheath in a type 1 diabetes patient. Cell 187 (22), 6152–6164. 10.1016/j.cell.2024.09.004 39326417

[B38] Wayne B. B. (2026). Process systems engineering in precision medicine: opportunities in autologous Car-T therapy. Eng. Life Sci. 26 (2), e70067. 10.1002/elsc.70067 41647312 PMC12871565

